# Molecular epidemiology of influenza B virus among hospitalized pediatric patients in Northern Italy during the 2015-16 season

**DOI:** 10.1371/journal.pone.0185893

**Published:** 2017-10-19

**Authors:** Antonio Piralla, Giovanna Lunghi, Luca Ruggiero, Alessia Girello, Sonia Bianchini, Francesca Rovida, Silvia Caimmi, Gian Luigi Marseglia, Nicola Principi, Fausto Baldanti, Susanna Esposito

**Affiliations:** 1 Molecular Virology Unit, Microbiology and Virology Department, Fondazione IRCCS Policlinico San Matteo, Pavia, Italy; 2 U.O.S Virology, IRCCS Fondazione Ca' Granda Ospedale Maggiore Policlinico di Milano, Milano, Italy; 3 Pediatric Highly Intensive Care Unit, Fondazione IRCCS Ca' Granda Ospedale Maggiore Policlinico and University of Milan, Milano, Italy; 4 Pediatric Clinic, University of Pavia, Fondazione IRCCS Policlinico San Matteo, Pavia, Italy; 5 Clinical, Surgical, Diagnostic and Pediatric Sciences, University of Pavia, Pavia, Italy; 6 Pediatric Clinic, Università degli Studi di Perugia, Perugia, Italy; George Mason University, UNITED STATES

## Abstract

**Background:**

The influenza B viruses belong to two lineages distinguished by their genetic and antigenic characteristics, which are referred to as the Yamagata and Victoria lineages, designated after their original isolates, B/Yamagata/16/88 and B/Victoria/2/87. The primary aim of this study was to evaluate the molecular characteristics of influenza B viruses circulating in a region of Northern Italy, Lombardia, during the influenza season of 2015–2016.

**Methods:**

Influenza B virus was detected using a respiratory virus panel of assays and an influenza B-specific real-time polymerase chain reaction. The complete influenza B hemagglutinin (HA) gene was amplified and sequenced directly from clinical specimens. Phylogenetic analysis was performed using nucleotide sequences.

**Results:**

A total of 71 hospitalized pediatric patients were influenza B positive. Phylogenetic analysis showed that the great majority of influenza B strains (66/71, 93.0%) belonged to the Victoria-lineage and were antigenically like vaccine strain (B/Brisbane/60/2008) included only in the quadrivalent vaccine. In the detected influenza B strains, a series of amino acid changes were observed in the antigenic regions: I117V, V124A, N129D, V146I, N197D, T199A, and A202T. However, only 2 amino acid changes were observed in the HA regions involved in receptor binding or in antibody recognition.

**Conclusions:**

All the influenza B strains identified in this study belonged to the influenza B Victoria lineage not included in the trivalent vaccine commonly used by the general population during the 2015–2016 influenza season in Italy. This indicates that protection against influenza B infection in the vaccinated population was in general very poor during the 2015–2016 influenza season.

## Background

Every year during influenza season, together with the two subtypes of influenza A virus (A/H1N1 and A/H3N2), two lineages of influenza B viruses (B/Victoria/2/87-like and B/Yamagata/16/88-like) simultaneously circulate and in some years are responsible for the major disease burden [1]. Unlike influenza A viruses, humans are the sole host of epidemiological relevance for influenza B viruses. This explains why antigenic shift does not occur in these viruses and they are not able to cause pandemics. However, influenza B viruses evolve mainly through genetic reassortment between strains of different lineages. This allows for escape from host immunity and preservation of the ability to cause disease [2]. Regardless of the lineage, influenza B infection can cause severe disease and death [1].

For many years, vaccine administration has been demonstrated as the best solution to reduce the influenza disease burden in humans [3, 4]. However, in the past, the recommended vaccines included only one influenza B lineage, chosen by the World Health Organization (WHO) based on surveillance data and using the lineage that had been observed to dominate in the previous year [5]. Only recently, quadrivalent vaccines containing both influenza B lineages have been prepared and licensed in some countries [6]. However, mainly because of the increased cost of vaccination, trivalent vaccines are still commonly used worldwide. It has been presumed that the prevalence of a given influenza B lineage may be driven by the virus contained in the trivalent vaccine, and the introduction of the quadrivalent preparation may reduce the selective pressure responsible for the modifications in lineage prevalence [7]. However, the relatively common emergence of influenza B re-assortants suggests that strict monitoring of the genetic characteristics of the circulating strains is necessary in order to assure the best selection of B viruses to be included in influenza vaccines. The primary aim of this study was to evaluate molecular characteristics of influenza B viruses circulating in a region of Northern Italy, Lombardia, during the influenza season of 2015–2016.

## Methods

### Study design

This study was carried out in the Pediatric High Intensive Care Unit, Fondazione IRCCS Ca' Granda Ospedale Maggiore Policlinico, Università degli Studi di Milano, Milan, Italy, and in the Pediatric Clinic and Section of Microbiology, Department of Clinical, Surgical, Diagnostic and Pediatric Sciences, University of Pavia, Pavia, Italy, between December 1, 2015 and March 31, 2016. Both institutions are located in Lombardia, a region of Northern Italy with approximately 10 million inhabitants. Nasopharyngeal swabs from all subjects, regardless of age and previous diagnosis of an underlying disease, presenting to the emergency room of both institutions for signs and/or symptoms of influenza-like illness (ILI) according to the definition of the Italian Ministry of Health [8], were collected and analyzed for respiratory viruses, including influenza. ILI is an acute respiratory disease of sudden onset, with fever (a temperature of >38°C) accompanied by at least one general symptom (i.e., headache, generalized malaise, a feverish sensation such as sweating and chills or asthenia) and at least one respiratory symptom (i.e., cough, pharyngodynia or nasal congestion). The respiratory symptoms had to have lasted at least three days to be considered in the analysis, and only one new-onset illness could be included in any two-week period. Data regarding influenza vaccination coverage were collected from parents. The study was approved by the Institutional Review Board of the Fondazione IRCCS Ca’ Granda Ospedale Maggiore Policlinico, Milan, Italy, and written informed consent was obtained from the enrolled subjects. For all the children, informed consent was signed by both parents or a legal guardian. Children ≥8 years were required to give their written assent before entering the study.

### Respiratory viruses detection

Respiratory samples were extracted using the QIAsymphony® instrument with QIAsymphony® DSP Virus/Pathogen Midi Kit (Complex 400 protocol) according to the manufacturer’s instructions (QIAGEN, Qiagen, Hilden, Germany). A panel of laboratory-developed real-time RT-polymerase chain reactions (PCR) or real-time PCRs was used to detect and quantify the following viruses: influenza virus A and B (including subtype determination), human rhinovirus (hRV), human parainfluenza virus (hPIV) 3, respiratory syncytial virus (RSV) types A and B, human coronavirus (hCoV)-OC43, -229E, -NL63, and -HKU1, and human metapneumovirus (hMPV) [9, 10]. Samples positive for influenza B were further analyzed for molecular characterization.

### Characterization of hemagglutinin (HA) gene of influenza B strains

The entire influenza B HA gene was amplified directly from clinical specimens using the SuperScriptIII One-Step RT-PCR amplification kit (Invitrogen, Carlsbad, USA) and the primers B/HA-f-6 5’-cacaaaatgaaggcaataattgtacta-3’ and B/HA-r+1730 5’-gacagatggagcawgaaacrttgtctctgg-3’ with the following cycling conditions: 50°C for 30 min, 95°C for 10 min; 50 cycles of 95°C for 30 s, 58°C for 30 s, and 72°C for 2 min; and a final extension cycle at 72°C for 5 min. Two semi-nested PCRs were performed (A: B/HA-f+33 5’-ggtagtaacatccaatgcagatcg-3’ B/HA-r+1294 5’-tatttcgttgtggagttcatccatggcacc3’; B: B/HA-f+1085 5’-aggaaaggggtttcttcggdgctattgctg-3’ B/HA-r+1730) with the following cycling conditions: 95°C for 10 min; 50 cycles of 95°C for 30 s, 59°C for 30 s; and 72°C for 1 min 30 s, with a final extension cycle at 72°C for 5 min.

Purified PCR products were sequenced using the BigDye Terminator Cycle-Sequencing kit (Applied Biosystems, Foster City, USA) and an ABI Prism 3130xl Genetic Analyzer (Applied Biosystems, Foster City, USA).

### Phylogenetic analyses

Sequences were assembled using Sequencher software, version 4.6 (Gene Codes Corporation, Ann Arbor, USA). Nucleotide alignments were constructed using the ClustalW program embedded in MEGA version 7 software [11]. Phylogenetic trees were generated by means of the Maximum likelihood method with the Kimura 3-parameter as an evolutionary model using MEGA version 7 software [12]. A bootstrap analysis with 1,000 replicates was performed. The nucleotide sequences identified in this study have been submitted to the GenBank database with accession numbers KY357920-KY357974.

### Selective pressure

In order to evaluate the HA evolution due to immunological pressure, a series of probabilistic models of codon substitution were used. In detail, tests for positive selection were conducted using single-likelihood ancestor counting (SLAC), fixed-effects likelihood (FEL), random-effects likelihood (REL), internal branch fixed-effects likelihood (IFEL), the mixed-effects model of evolution (MEME), and fast unconstrained Bayesian approximation (FUBAR) on the Datamonkey server [13], with the *dN/dS* ratios calculated using the SLAC and FEL codon-based maximum likelihood approaches. SLAC counts the number of non-synonymous changes per non-synonymous site (*dN*) and tests whether it is significantly different from the number of synonymous changes per synonymous site (*dS*). FEL estimates the ratios of non-synonymous to synonymous changes for each site in an alignment [14]. The IFEL method is similar to FEL, but tests for site-by-site selection only along internal branches of the phylogeny. To avoid an excessive false-positive rate, sites with SLAC, FEL, IFEL and MEME *p*-values of <0.1 and a FUBAR posterior probability of >0.90 were accepted as candidates for selection.

## Results

### Study population

A total of 763 children (416 males, 54.5%; median age, 4.3 years; age range, 3 months to 14 years) with ILI were enrolled, and their nasopharyngeal samples were collected. We obtained 523 samples from Milano and 240 from Pavia. Among them, 137 (17.9%) tested positive for influenza viruses, 66 (48.2%) for influenza A and 71 (51.8%) for influenza B. None of the enrolled children had received the seasonal influenza vaccine prepared for the 20015–2016 season.

### Phylogenetic analyses

A total of 55/71 (78.9%) influenza B strains were sequenced. From 49/55 (90.1%), nearly complete HA gene sequencing was performed, while for 6/55 (10.9%) strains, only a partial HA sequence, ranging from 597 nt to 1006 nt, was available. No sequencing data were obtained for 16/71 (21.1%) patients. Of these, 11/16 (68.8%) were due to the low viral load, while no residual samples were available from 5/16 (31.2%). The overall nucleotide identity of analyzed HA sequences with the reference influenza B vaccine strain (B/Brisbane/60/2008) ranged between 98.5% and 99.8%, with a maximum of 22 nucleotides and 6 amino acid changes.

**Fig 1:**
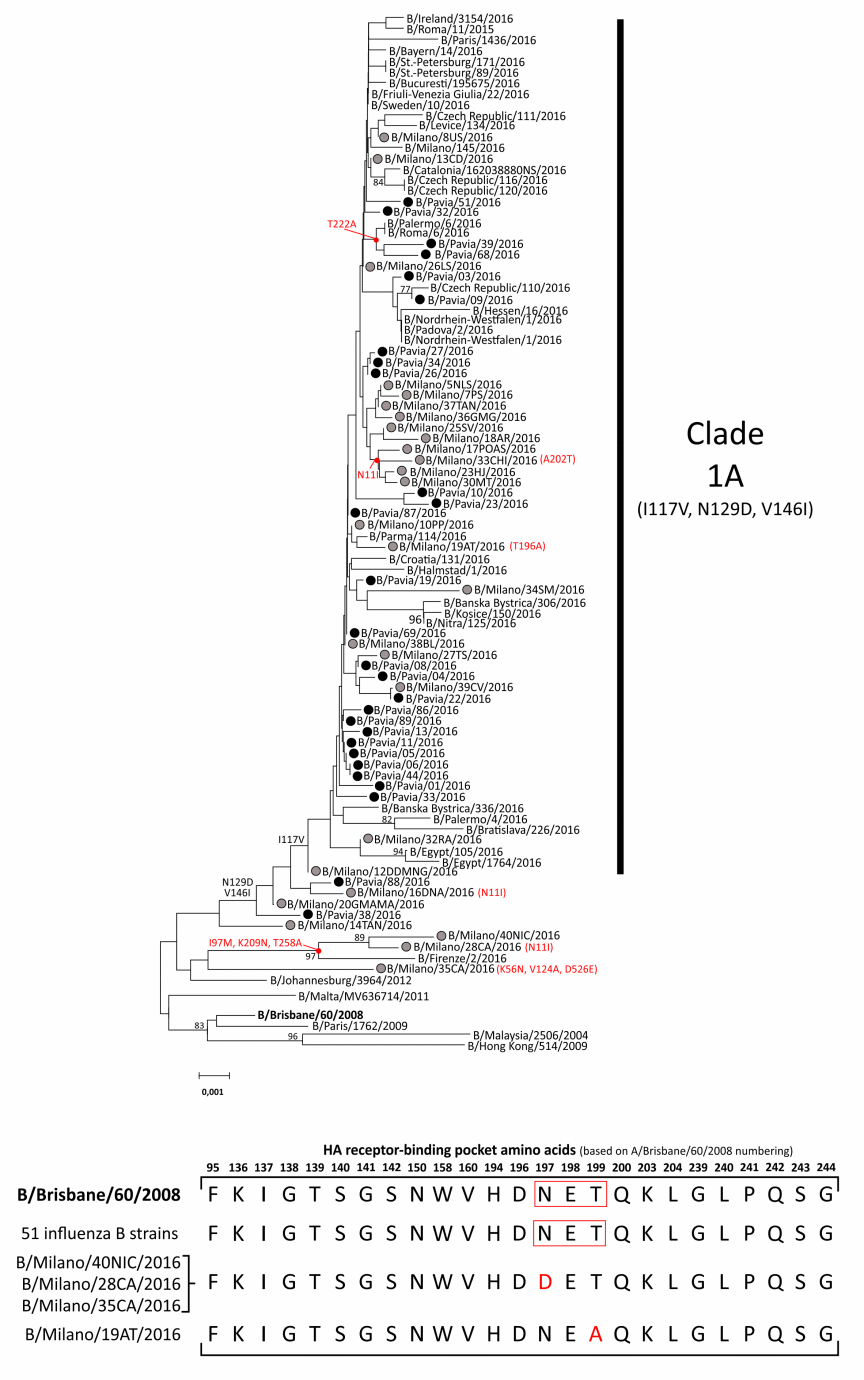
(A) The evolutionary history was inferred by using the Maximum Likelihood method based on the Tamura Nei model. The bootstrap consensus tree inferred from 1000 replicates is taken to represent the evolutionary history of the analyzed taxa. The percentage of replicate trees in which the associated taxa clustered together in the bootstrap test (1000 replicates) is shown next to the branches. The Influenza B sequences originally found in this study are reported with gray circles (Milan) and black circles (Pavia). (B) Sequence alignment of the influenza B HA receptor-binding pocket amino acids. The glycosylation site in the sequence of B/Brisbane/60/2008 as well as in other influenza B strains is boxed in red.

Phylogenetic analysis showed that the great majority of influenza B strains (47/55, 85.5%) fall in a clade defined by amino acid substitutions I117V, N129D and V146I (based on B/Brisbane/60/2008 numbering; Fig 1A). However, this clade remains antigenically like the vaccine strain only included in the quadrivalent vaccine. Of note, three strains were closely related to “old” influenza B strains circulating in previous seasons. Two of them, B/Milano/40NIC/2016 and B/Milano/28CA/2016, were characterized by I97M, K209N, and T258A mutations, as also observed in another Italian influenza B strain (B/Firenze/2/2016) circulating in the same period. The B/Milano/35CA/2016 strain was characterized by the K56N, V124A and D526E mutations (Fig 1A).

### Protein sequence and selective pressure analyses

In the studied influenza B strains, a series of amino acid changes were observed: I117V, V124A, N129D, V146I, D197N, T199A, and A202T (Table 1). However, only 2 amino acid changes were observed in HA regions involved in the receptor binding or in the antibody recognition [the 120 loop (position 116–137), the 150 loop (141–151), the 160 loop (162–167) and the helix 190 (194–202) [15] (Fig 1B). In detail, B/Milano/40NIC/2016, B/Milano/28CA/2016 and B/Milano/35CA/2016 harbored the N197D substitution, while the B/Milano/19AT/2016 strain was characterized by a different amino acid change, T199A, highlighted in red in Fig 1B. Interestingly, these changes led to the loss of a glycosylation site near the receptor-binding region (helix-190).

**Table 1:** Amino acid mutations in the antigenic sites of the influenza B hemagglutinin (HA) gene.

Subunit	Epitope (residue location)	Mutation (number of strains)
HA1	120-loop (116–137)	I117V (42), V124A (1), D129N (44)
	150-loop (141–150)	I146V (3)
	160-loop (162–167)	-
	190-loop (194–202)	D197N (46), T199A (1), A202T (1)

A global analysis of selective pressure made using the SLAC model indicated an estimated overall *dN/dS* ratio of 0.175. A total of 23 codons were observed with at least one amino acid change compared to the vaccine strain. Overall, no sites were identified as being under positive selection by site-specific analyses in the influenza B alignment by at least three of the methods used (SLAC, FEL, REL, FUBAR and MEME) (Table 2). The IFEL model used to determine the selection pressure acting on the codons along the internal branches of the tree identified a positively selected codon in the influenza B sequences (Table 2). In fact, the N11I change was observed in six influenza B strains; four of them (B/Milano/17POAS/2016, B/Milano/33CHI/2016, B/Milano/23HJ/2016, and B/Milano/30MT/2016) were clustered together in a separate branch of the tree as shown in Fig 1A. In contrast, a series of negative selected sites were identified (Table 2). Nearly half of them were within the HA2 region not strictly correlated with surface exposition.

**Table 2:** Positive and negative selected sites for influenza B strains identified in this study.

Methods	Flu B codons	
	Positive selection	Negative selection
SLAC	none	**80**, 210, **244**, **256,** 461, 519, 532, 549, 550, 556, 559, 562, **563**
FEL	none	**73**, 78, **80**, 122, **244**, **256**, **265**, 297, **351, 358, 380**, 462, **531, 563**, 565
REL	**11**, 58, 117, 197, 526	**73**, **80**, **244**, **256, 265**, **351, 358, 380**, **531**, **563**
FUBAR	**11**	**73**, 78, **80**, 122, **244**, **256,265**, 297, **351, 358, 380**, 462, **531, 563**, 565.
MEME	none	none
IFEL	**11**	80, **244, 256, 265**, **351**, 563

FEL: fixed-effects likelihood; FUBAR: fast unconstrained Bayesian approximation; IFEL: random-effects likelihood; MEME: mixed-effects model of evolution; REL: internal branch fixed-effects likelihood; SLAC: single-likelihood ancestor. Positions selected by at least 3 methods are reported in bold.

## Discussion

This study enrolled children with ILI. During winter season, several respiratory viruses circulate. Preschool children, as most of those enrolled in this study, are highly susceptible to all these infectious agents and have clinical manifestations quite similar to those of influenza. Consequently, the relatively low percentage of patients with laboratory-documented influenza does not surprise. On the other hand, during the 2015–2016 influenza season, the prevalence of influenza among children aged 5–14 years in Italy was calculated to be 17.8% [16], a value quite similar to the found in this study.

Antigenic characterization of influenza B strains is important in the assessment of the similarity between reference viruses and circulating viruses. In addition, these characterizations are also useful annually to the WHO to recommend which influenza strains should be included in the vaccine for the Northern hemisphere. Based on phylogenetic data, all the influenza B strains circulating in the two children’s hospitals belonged to the B/Brisbane/60/2008 clade within the B/Victoria-lineage. This finding is similar to the epidemiological scenario observed during the last influenza season by sentinel sources of the European Center for Disease Control (ECDC) [17] and the Italian National Influenza Center [18], that reported an incidence of 85.9% and 95.0%, respectively, for strains belonging to the B/Victoria-lineage. Unfortunately, during the 2015–2016 season the B/Victoria-lineage strain was not included in the trivalent vaccine licensed for the Northern hemisphere worldwide. According to WHO recommendation a B/Phuket/3073/2013-like virus (B/Yamagata lineage) was included.\\ Therefore, this finding could be the reason for the high circulation of B/Victoria-lineage strains in Italy as well as throughout some other European countries. Data collected by the ECDC [17] clearly indicate that besides Italy, also in Belgium, France, and Luxembourg, B virus was the predominant cause of influenza. In the whole Italy prevalence of B influenza virus was 57% [18], a value not substantially different from that found in this study. Based on these epidemiological data, the WHO has changed the recommendation for the 2016–2017 influenza trivalent vaccine composition for the Northern hemisphere [5]. In contrast, the use of a quadrivalent vaccine containing two influenza B strains could reduce the mismatch between vaccine and circulating influenza strains. Although a quadrivalent influenza vaccine is licensed in Italy for subjects ≥3 years, its use is still restricted, mainly because it is not definitively established if the introduction of this more expensive vaccine is really cost/effective. Among the detected B strains, three were characterized by a different pattern of mutations. Two of them harbored the I97M, K209N and T258A mutations, while one strain harbored the K56N, V124A and D526E mutations. These strains belong to two out of three B/Brisbane/60/2008 subclades previously described to circulate in Australia in 2015 [19]. In detail, as reported by Barr and colleagues [19], the time to the most recent common ancestor calculated for these three subclades was summer 2013. This finding suggests a multiple introduction of different influenza B strains during the last influenza season in the Lombardy region.

The evolution of the influenza B virus is driven by mutations on the membrane-distal domain of HA1 in specific domains such as loop-120, loop-150, loop-160 and helix 190 [20]. Several mutations have been observed in these specific regions, but the great majority of them were characteristic of the circulating strains (i.e., I117V, N129D and V146I), while the other mutations were observed in a few influenza strains as only sporadic occurrences. In four influenza B strains, a loss of an important glycosylation site in position 194–196 (HA1) was observed. In an experimental egg-adapted influenza B variant, the absence of glycans at residue 194 was observed [21]. In fact, these influenza virus variants have been observed to grow better in chicken eggs than mammalian virus variants of the same origin [21]. This finding suggests a possible impact of this loss in the antibody recognition after vaccination.

The analysis of the ratio of the number of non-synonymous and synonymous substitutions per site (*dN/dS*) revealed a lower level of evolutionary dynamics compared to influenza A/H1N1 and A/H3N2 [22]. However, our data are in accordance with the recent publication of Vijaykrishna et al. that reported a *dN/dS* ratio of 0.19 for the Victoria-lineage viruses [23]. An overall analysis of selective pressure within the influenza strains sequenced here revealed only one site under positive selection and a series of codons under purifying selection. This finding suggests a moderate immune selection in the Victoria-lineage of influenza B virus persistently circulating in humans. In addition, it has also shown that influenza B evolution could be driven by both positive and negative selection. This moderate evolution of circulating influenza B strains makes these strains antigenically similar to the vaccine strain. Indeed, since the 2009–2010 season, the influenza B vaccine strain, B/Brisbane/60/2008, has not been changed [5]. These data, together with the antigenic characterizations performed by ECDC using hemagglutinin inhibition (HI), showed an HI reactivity pattern for the circulating influenza B strains. These data confirm the efficacy of the influenza vaccine on the protection against influenza B circulating strains. However, based on epidemiological data from this study, it would be more appropriate to use of the quadrivalent instead of the trivalent vaccine.

In conclusion, influenza B viruses that circulated in Lombardy Region of Italy during the influenza season 2015–2016 belonged to the influenza B Victoria-lineage not included in the trivalent vaccine commonly administered during the 2015–2016 influenza season in Italy. The great majority of influenza B strains were similar to those circulating in Europe in the same period. Three strains were closely related to “old” influenza B strains circulating in previous seasons. All these findings indicate that in Europe, and in particular in the Lombardy Region of Italy, protection against influenza B infection in vaccinated subjects might have been very poor during that period. The introduction of the quadrivalent vaccine in the Italian vaccination campaign could prevent or reduce the circulation of both lineages of influenza B and improve the protection of the population against a potential severe disease.

## Ethics approval and consent to participate

This study was approved by the Ethics Committee of the Fondazione IRCCS Ca’ Granda Ospedale Maggiore Policlinico, Milan, Italy. Written informed consent was obtained from either the parent(s) or legal guardian(s) of each study participant as well as from children aged ≥8 years.

## Abbreviations

ECDC: European Center for Disease Control
FEL: fixed-effects likelihood
FUBAR: fast unconstrained Bayesian approximation
hCoV: human coronavirus
HI: hemagglutinin inhibition
hMPV: human metapneumovirus
hPIV: human parainfluenza virus
hRV: human rhinovirus
IFEL: internal branch fixed-effects likelihood
ILI: influenza-like illness
MEME: mixed-effects model of evolution
PCR: polymerase chain reaction
REL: random-effects likelihood
RSV: respiratory syncytial virus
SLAC: single-likelihood ancestor counting
WHO: World Health Organization
